# Bewegungsförderung in der Ganztagsschule – Herausforderungen und Chancen

**DOI:** 10.1007/s00103-026-04315-6

**Published:** 2026-10-12

**Authors:** Nils Neuber, Miriam Kehne

**Affiliations:** 1 Institut für Sportwissenschaft, Universität Münster, Münster, Deutschland; 2 Department Sport und Gesundheit, Universität Paderborn, Paderborn, Deutschland

**Keywords:** Ganztagsschule, Bewegungsförderung, Bewegungserziehung, Forschungsstand, Qualitätsdimensionen, All-day school, Physical-activity promotion, Movement education, State of research, Quality dimensions

## Abstract

Durch die Einführung von Ganztagsschulen verbringen Schülerinnen und Schüler heute deutlich mehr Zeit in der Schule als im 20. Jahrhundert. Damit ist eine Zunahme der öffentlichen Verantwortung für das Aufwachsen junger Menschen verbunden. Einerseits können dadurch mehr Heranwachsende pädagogisch erreicht werden, insbesondere Kinder aus sozial herausfordernden Lagen. Andererseits verändert sich das Freizeitverhalten der jungen Generation, nicht zuletzt hinsichtlich ihrer Bewegungs‑, Spiel- und Sportaktivitäten. Ganztagsschulen kommt damit zunehmend die Aufgabe zu, adäquate Bewegungsmöglichkeiten für Heranwachsende anzubieten.

Vor diesem Hintergrund befasst sich der Beitrag mit den Herausforderungen und Chancen des Ganztags für eine umfassende Bewegungsförderung von Kindern. Ausgehend von einer pädagogischen Konkretisierung des Begriffs der Bewegungsförderung werden *angeleitete* und *nichtangeleitete* Bewegungsaktivitäten in der offenen Ganztagsgrundschule differenziert betrachtet. Anschließend wird der Forschungsstand zur Bewegungsförderung im Ganztag zusammengefasst und es werden Qualitätsdimensionen für Bewegungs‑, Spiel- und Sportangebote umrissen. Abschließend wird für eine systematische Verzahnung formeller, nichtformeller und informeller Bewegungsaktivitäten plädiert, wobei nichtangeleiteten Bewegungsaktivitäten im Sinne der Alltagsaktivität von Kindern besondere Bedeutung zukommt.

## Abstract (en)

With the introduction of all-day schools, pupils now spend considerably more time at school than they did in the twentieth century. This is associated with increased public responsibility for the development of young people. On the one hand, this means that more young people can be reached through educational provision, particularly children from socially disadvantaged backgrounds. On the other hand, the leisure behaviour of the younger generation is changing, not least in terms of their physical activity, play and sporting pursuits. Consequently, all-day schools are increasingly tasked with providing adequate opportunities for physical activity for young people.

Against this background, this article examines the challenges and opportunities of all-day schooling for the comprehensive promotion of physical activity among children. Beginning with an educationally grounded clarification of the concept of physical activity promotion, the article examines *guided* and *unguided* movement activities in open all-day primary schools, drawing distinctions between them. The article then summarises the current state of research on physical activity promotion in all-day settings and outlines quality dimensions for physical activity, play and sport programmes. Finally, it advocates for the systematic integration of formal, non-formal and informal physical activities, emphasising the particular importance of unguided physical activity as part of children’s everyday lives.

## Einleitung

Im 20. Jahrhundert war die Schule in Westdeutschland im Wesentlichen eine Halbtagsschule: Vormittags gingen Kinder und Jugendliche zur Schule, nachmittags machten sie Hausaufgaben und nahmen ansonsten an außerschulischen Freizeitangeboten teil oder gingen selbstorganisierten Freizeitaktivitäten nach. Erst der PISA-Schock zu Beginn des 21. Jahrhunderts mit den schlechten schulischen Leistungen deutscher Schülerinnen und Schüler im internationalen Vergleich führte im Zuge struktureller Bildungsreformen zu einer flächendeckenden Einführung von Ganztagsschulen in Deutschland. Dabei werden 3 Formen unterschieden: In gebundenen Ganztagsschulen nehmen alle Schülerinnen und Schüler am Ganztagsangebot teil, das im Wesentlichen von akademisch gebildeten Lehrkräften geleitet wird. In teilweise gebundenen Ganztagsschulen gilt dies nur für einen Teil der Schülerinnen und Schüler; auch hier werden die Angebote zumeist von Lehrkräften durchgeführt. In offenen Ganztagsschulen können einzelne Schülerinnen und Schüler unabhängig von ihrer Klassenzugehörigkeit teilnehmen. Während der Unterricht am Vormittag von Lehrkräften erteilt wird, gestalten pädagogische Fachkräfte in der Regel die Angebote am Nachmittag. Offene Ganztagsschulen bilden in Deutschland die größte Gruppe. Darunter wiederum sind offene Ganztags*grund*schulen mit Abstand am häufigsten vertreten [1], weshalb der Fokus im Folgenden auf diese Schulform gelegt wird.

Der Ausbau der Ganztagsschulen war von Anfang an mit anspruchsvollen Zielsetzungen verbunden. An erster Stelle stand die Forderung nach einer individuellen Förderung aller Schülerinnen und Schüler [2]. Damit verbunden ist die Idee einer veränderten Lernkultur, die durch eine bessere Rhythmisierung des Schulalltags ein effizienteres Lernen ermöglichen, zugleich aber auch mehr Partizipation und soziales Lernen in die Schule bringen soll [3]. Auch die Idee der Chancengerechtigkeit im Bildungssystem ist ein zentrales Ziel der Ganztagsschule. Schließlich zielt die Ganztagsschule auf eine bessere Vereinbarkeit von Familie und Beruf. Während diese familienpolitische Zielsetzung zu großen Teilen umgesetzt werden konnte, wurden die pädagogischen Zielsetzungen bislang nur in Ansätzen erreicht. So verfolgt mehr als die Hälfte der Ganztagsschulen nach eigenen Angaben keine Ziele der Kompetenzorientierung oder Begabungsförderung. Die Betreuung steht im Vordergrund, nicht aber die individuelle Förderung der Kinder oder eine Veränderung der Lernkultur [4].

Aktuell befinden sich knapp 72 % aller Schulen in Deutschland im Ganztagsbetrieb und rund 57 % der Schülerinnen und Schüler nehmen am Ganztag teil [1]. Mit Blick auf den im Sommer 2026 mit Klasse 1 eingeführten Rechtsanspruch auf ganztägige Förderung in der Grundschule ist davon auszugehen, dass diese Zahlen weiter steigen werden. Schülerinnen und Schüler verbringen damit deutlich mehr Zeit in der Schule als zuvor. Das führt zu einer stärkeren Strukturierung ihres Lebensalltags und damit letztlich zu einer Zunahme öffentlicher Verantwortung für das Aufwachsen von Kindern und Jugendlichen [5]. Das hat unmittelbare Auswirkungen auf das Freizeitverhalten junger Menschen, nicht zuletzt auf ihre Bewegungs‑, Spiel- und Sportaktivitäten. Wenn Kinder und Jugendliche am Nachmittag weniger in Sportvereinen oder selbstorganisiert aktiv sind, kommt der Ganztagsschule zunehmend die Aufgabe zu, adäquate Bewegungsmöglichkeiten und -angebote für Heranwachsende zu organisieren. Zugleich erreicht die Ganztagsschule aber auch junge Menschen, die bislang nicht sportlich aktiv waren – im Sinne der Chancengerechtigkeit ist das durchaus positiv zu bewerten.

Vor diesem Hintergrund zielt der Beitrag darauf ab, zu klären, welche Chancen die Ganztagsschule für die Bewegungsförderung von Kindern bietet, aber auch welche Herausforderungen damit verbunden sind. Dazu wird zunächst der Begriff der Bewegungsförderung konkretisiert. Auf dieser Grundlage werden Bewegungs‑, Spiel- und Sportaktivitäten im Ganztag charakterisiert und mit aktuellen Befunden zu Sichtweisen von Kindern und pädagogischem Personal illustriert, bevor abschließend mögliche Qualitätsdimensionen für Sportangebote skizziert und Perspektiven für die Bewegungsförderung im Ganztag entwickelt werden.

## Bewegungsförderung als pädagogischer Begriff

Im pädagogischen Feld wurde lange Zeit vor allem der Begriff der Bewegungs*erziehung *verwendet. Das *Handbuch der Bewegungserziehung* von Renate Zimmer erschien erstmals 1993. Darin wurde ausführlich begründet, warum und wie pädagogisch verantwortete Bewegungsangebote die Entwicklung von Kindern fördern können [6]. Viele weitere Ansätze bedienten sich der Argumentationsfigur der Bewegungserziehung, etwa im Bereich der ästhetischen Erziehung [7], der Grundschulpädagogik [8] oder der Bewegungspädagogik [9]. So unterschiedlich die Ansätze sind, geht es dabei immer um eine normative Begründung der Bewegungserziehung, die als umfassende motorische, emotionale, soziale und kognitive Förderung von Kindern begriffen wird. Bewegung und Spiel werden darin als elementare Betätigungs- und Ausdrucksformen von Kindern verstanden, denen zahlreiche Funktionen zugeschrieben werden, z. B. personale, soziale, explorative, expressive oder adaptive Funktionen [6]. Damit verbunden ist die Frage, welchen Sinn Kinder ihrer Bewegungsaktivität zuschreiben. Dieses weitgefasste Verständnis von Bewegung bzw. Bewegungserziehung bildet bis heute die Grundlage für die Grundschuldidaktik im Sport und ist damit auch maßgeblich für pädagogisch verantwortete Bewegungsangebote im Ganztag [10].

Parallel zum sportpädagogischen Konzept der Bewegungserziehung entwickelte sich der Ansatz der Bewegungs*förderung*. Das Handbuch *Bewegungsförderung in der Schule* von Sigrid Dordel erschien erstmals 1987. Von Beginn an wurde dieser Ansatz funktional bzw. medizinisch ausgelegt [11]. In diesem Bereich hat der Begriff bis heute seine stärkste Bedeutung, was eine Studie des Bundesgesundheitsministeriums mit dem Titel „Bewegungsförderung bei Kindern und Jugendlichen in Deutschland“ bestätigt [12]. Bewegungsaktivitäten junger Menschen werden darin vor allem biomedizinisch begründet und orientieren sich zumeist an der Vorgabe bestimmter Bewegungsumfänge, die gut messbar sind, nicht aber an konkreten Bewegungsqualitäten, wie selbstwertdienliche Könnenserlebnisse oder soziale Interaktionen. So heißt es in den nationalen Empfehlungen für Bewegung und Bewegungsförderung: Bewegung entspricht „der durch die Skelettmuskulatur erzeugten Bewegung des Körpers, die zu einem substanziellen Anstieg des Energieverbrauchs über den Ruheenergieverbrauch“ hinausführt [13, S. 19]. Im medizinisch-gesundheitswissenschaftlichen Feld ist dieses funktionale Verständnis von Bewegung bzw. Bewegungsförderung bis heute maßgeblich (z. B. [14]).

Trotz dieser unterschiedlichen Sichtweisen wird der Begriff der Bewegungsförderung zunehmend auch im sportpädagogischen Kontext gebraucht, etwa in der Kindertagesstätte [15], in der Schule [16] oder im Ganztag [17]. Für diese pädagogischen Settings muss geklärt werden, wie der Begriff verstanden werden soll, damit er in der Praxis wirksam werden kann. Geht es um eine biomedizinische Sicht, die ausschließlich Bewegungsumfänge definiert, werden Kinder in diesen Feldern nur schwer erreicht, weil sie kein ausgeprägtes Gesundheitsmotiv haben. Das liegt vor allem daran, dass sie vergleichsweise wenig Beschwerden haben und sich trotz zunehmender psychophysischer Belastungen weitgehend gesund fühlen [18]. Versteht man Bewegungsförderung dagegen als umfassenden Begriff, der verschiedene Funktionen von Bewegung einbezieht und den subjektiven Sinn, den Kinder im Bewegungsspiel sehen, mit aufgreift, können Maßnahmen im pädagogischen Feld wirksam werden. Ähnlich argumentiert auch der Doppelauftrag des Schulsports, der mit der Erziehung zum Sport und durch Sport sowohl motorische Aspekte als auch übergreifende Fähigkeiten, etwa im emotionalen oder sozialen Bereich, zum Ziel hat. Auch Konzepte der Bewegungsförderung im Ganztag greifen darum sowohl auf gesundheitliche als auch auf pädagogische Begründungsmuster zurück und konzipieren Bewegungs‑, Spiel- und Sportaktivitäten entsprechend mehrperspektivisch [19]. Für Bewegungsaktivitäten von Kindern im Ganztag sind damit beide Sichtweisen relevant.

## Bewegungs‑, Spiel- und Sportaktivitäten im offenen Ganztag

**Abb. 1:**
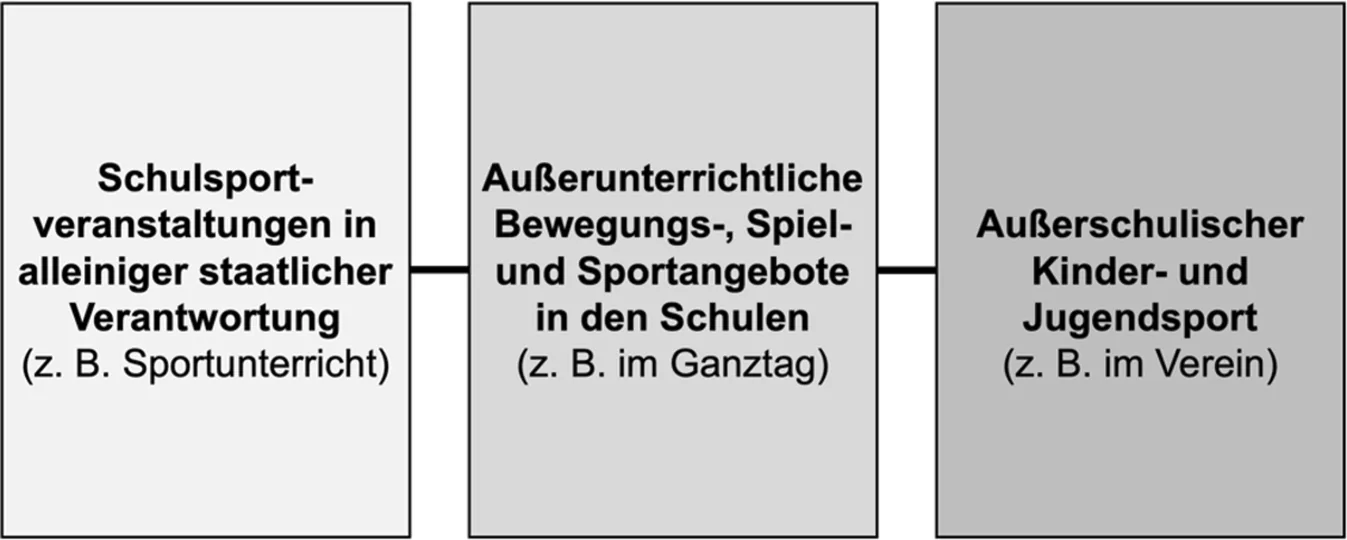
Drei-Säulen-Modell der Kinder- und Jugendbildung im Sport (modifiziert nach Pack und Bockhorst 2011, S. 169 [22])

Der Nachmittag in offenen Ganztagsgrundschulen wird in der Regel in die Bereiche Mittagessen, Hausaufgabenbetreuung und Freizeit unterteilt. Bewegungsaktivitäten werden dem sogenannten Freizeitbereich zugeordnet. Dabei geht es um mehr „als nur um eine nahtlose Verlängerung von Vereinsangeboten oder nur um eine Verdoppelung des schulischen Sportunterrichts“ [20, S. 21]. Vielmehr handelt es sich um einen neuen Angebotstyp im Schnittfeld von Sport- und Sozialpädagogik bzw. formellem und informellem Lernen, der zwischen Sportunterricht und Vereinstraining angesiedelt werden kann [21]. Insgesamt hat sich damit zwischen dem staatlich verantworteten Schulsport und dem gemeinwohlorientierten Vereinssport „im Bereich der außerunterrichtlichen Angebote eine weitere ‚Säule‘ der Kinder- und Jugendbildung im Sport gebildet“ ([22, S. 169]; Abb. 1).

Die Bewegungsangebote finden sowohl angeleitet im Rahmen von Arbeitsgemeinschaften oder Kursen als auch nichtangeleitet im Sinne eines freien Bewegungsspiels, z. B. auf dem Schulhof, statt. Während die Arbeitsgemeinschaften von den Kindern gewählt werden können, findet das freie Bewegungsspiel weitgehend ungeregelt statt. Insofern bietet der Ganztag besondere Potenziale für eine kindgerechte Bewegungsförderung im Schulalltag. Allerdings bleiben Vormittags- und Nachmittagsbereich in offenen Ganztagsgrundschulen weitgehend voneinander getrennt, sodass sich auch die Strukturierung des Vormittags nicht grundsätzlich verändert. Zugleich können am Nachmittag zwar viele Kinder erreicht werden, die Teilnahme an den Angeboten ist jedoch nicht verpflichtend.

### Angeleitete Angebote.

Charakteristisch für angeleitete Ganztagsangebote im Sport sind u. a. fehlender Notendruck, kaum wettkampfsportliches Engagement, heterogene, oft jahrgangsübergreifende Gruppenzusammensetzung sowie die Anleitung durch pädagogische Fachkräfte [21]. Dabei kann zwischen sportartspezifischen und sportartübergreifenden Aktivitäten unterschieden werden. Je nach Studie liegen die Anteile zwischen 52 % und 71 % für die sportartspezifischen und 25 % und 37 % für die sportartübergreifenden Angebote. Insgesamt bedeutet das etwa ein Verhältnis von 2:1 [23, S. 40].

Bei den sportspezifischen Angeboten dominieren in allen Stichproben die Ballsportarten, wobei Fußball – wenig überraschend – am häufigsten angeboten wird. Es folgen Rückschlagspiele, Tanzen, Turnen und Kampsportarten, wobei die Angaben je nach Studie variieren [24]. Für sportartübergreifende Angebote sind verlässliche Aussagen schwieriger, da die verwendeten Kategorisierungen uneinheitlich sind. So unterscheidet eine niedersächsische Studie Bewegungsförderung/Breitensport, Tanzsport, Gesundheitssport sowie Abenteuer- und Erlebnissport [25, S. 21–22].

Auffällig ist zudem, dass bislang kaum Erkenntnisse zur methodischen Inszenierung der Sportangebote im Ganztag vorliegen. Offen ist etwa, ob sich die Angebote stärker an der Sache Sport orientieren und entsprechend eher deduktiv geschlossen vermittelt werden oder ob die Bedürfnisse und Wünsche der Kinder im Vordergrund stehen und die Angebote eher induktiv offen gestaltet werden [26]. Die Art und Weise der Vermittlung, die Ausgestaltung des neuen Angebotstyps „Sport im Ganztag“, wurde bislang nicht untersucht. Dies ist insbesondere im Hinblick auf die Qualität der Angebote relevant, da die Partizipation der Kinder nicht nur ein zentrales pädagogisches Ziel darstellt, sondern zugleich ihre Motivation zur Bewegung fördern kann.

### Nichtangeleitete Angebote.

Neben den angeleiteten Bewegungs‑, Spiel- und Sportangeboten gibt es das Feld der informellen Bewegungsaktivitäten von Kindern in Pausen oder offenen Spielzeiten. Tatsächlich kommt dem freien, nichtangeleiteten Bewegungsspiel im Ganztag eine besondere Bedeutung zu. Im offenen Ganztag haben Kinder bis zu 20 h frei verfügbarer Zeit pro Woche [27]. Wenn es gelingt, den schulischen Raum bewegungsfreundlich zu gestalten, kann damit nicht nur dem kindlichen Spiel- und Bewegungsdrang Rechnung getragen, sondern auch ein hohes Maß an Alltagsaktivität gefördert werden.

Arbeiten zur Bewegten Schule betonen entsprechend die Bedeutung einer bewegungsfreundlichen Gestaltung des Schulgeländes. Neben Sportgelegenheiten wie Basketballkörben, Tischtennisplatten und Fußballtoren können beispielsweise auch Balancierstämme, Hüpfsteine, Kletterwände, Hangelpfade, Wasserläufe, Spielgebüsche, Minigärten oder Freiluftklassen vielfältige Bewegungsanreize bieten [28]. Neben dem Schulgelände und der Sporthalle als typische Bewegungsorte kann auch eine bewegungsfreundliche Gestaltung des Schulgebäudes zu informellen Bewegungs- und Spielaktivitäten anregen. Für die Ganztagsschule hat sich dafür der Begriff der „Schulfreiräume“ etabliert, also der Orte, an denen Kinder ihre freie Zeit verbringen, an denen sie sich treffen und spielen, aber eben auch bewegen können. Diese informellen Bewegungsmöglichkeiten sollten im Hinblick auf ihre gesundheitsbezogenen Wirkungen nicht unterschätzt werden, was erste Analysen bestätigen [29].

## Forschungsstand zur Bewegungsförderung im Ganztag

Mit dem Ausbau von Ganztagsschulen hat sich ein Forschungsfeld zu Bewegung, Spiel und Sport im Ganztag entwickelt. Ein aktuelles Scoping-Review gibt einen Überblick über die Forschung der vergangenen knapp 20 Jahre [30]. Diese ist durch verschiedene inhaltliche Perspektiven, Studiendesigns, Erhebungsinstrumente und Untersuchungsgruppen sowie einen hohen Anteil regionaler Querschnittsstudien gekennzeichnet. Aufgrund dieser methodischen Heterogenität sind die Studienergebnisse nur eingeschränkt vergleichbar und übertragbar. Hinzu kommt, dass sich die Konzepte und die Organisation des Ganztags zwischen und innerhalb der Bundesländer zum Teil erheblich unterscheiden. Zudem stammt ein Großteil der Studien aus den Anfangsjahren der Ganztagsschulentwicklung und bildet die heutige Situation daher nur eingeschränkt ab. Die wenigen größeren Studien liegen mittlerweile mehr als 10 Jahre zurück, sodass eine verlässliche und umfassende Beschreibung der aktuellen Situation kaum möglich ist. Auf Basis dieses wenig konsistenten und veralteten Forschungsstandes ist die Ableitung fundierter Handlungsperspektiven für Bewegung, Spiel und Sport im Ganztag kaum möglich. Dennoch gibt es einige zentrale Erkenntnisse [30].

Einheitlich zeigen Studienergebnisse, dass Bewegungs‑, Spiel- und Sportangebote die häufigsten im schulischen Ganztag sind. Schon vor mehr als 10 Jahren konnten über 50 % der Kinder im Ganztag mit Bewegungsangeboten erreicht werden [31–33]. Zumeist werden die Angebote in Kooperationen mit schulexternen Partnern durchgeführt, am häufigsten mit Sportvereinen [34]. Allerdings mangelt es häufig an systematischen Austauschprozessen und einer strukturellen Einbindung der Kooperationspartner [33, 35–38]. Wie die Kooperationen sowie Bewegungs‑, Spiel- und Sportaktivitäten im Ganztag konkret gestaltet und umgesetzt werden, ist abhängig von den jeweiligen schulischen Rahmenbedingungen. Herausforderungen bestehen insbesondere bei der konzeptionellen Einbindung sowie hinsichtlich der finanziellen, räumlichen und personellen Ressourcen [4, 39]. Hinzu kommt, dass das im Ganztag tätige Personal über unterschiedliche Qualifikationen verfügt [40, 41]. Dabei bestehen sowohl zwischen den Bundesländern als auch zwischen einzelnen Schulen Unterschiede. Mitunter wird eine grundlegende Qualifikation, etwa die Übungsleiter-C-Lizenz im 3‑stufigen Lizenzausbildungssystem des Deutschen Olympischen Sportbunds, als Mindestqualifikation gefordert. Es gibt aber auch Bundesländer, die keine Mindeststandards definieren [31, 38, 42, 43].

Eine aktuelle qualitative Studie zu Bewegungsangeboten an Ganztagsgrundschulen ergänzt die Ergebnisse des oben genannten Scoping-Reviews [30] um die Perspektiven von Kindern und pädagogisch tätigem Personal [44, 45]. In der Teilstudie des vom Bundesministerium für Bildung, Familie, Senioren, Frauen und Jugend (BMFSFJ) geförderten Projekts „Move For Health“ standen 2 übergeordnete Fragestellungen im Fokus: „Wie ist der Status quo von Bewegung, Spiel und Sport im schulischen Ganztag aus Sicht des pädagogischen Personals und der Kinder?“ Und: „Welche Herausforderungen, Bedarfe und Wünsche in Bezug auf Bewegung, Spiel und Sport im schulischen Ganztag bestehen aus Sicht des pädagogischen Personals und der Kinder?“ Die qualitativen Daten wurden in insgesamt 52 Interviews mit 140 Personen, in 8 Fokusgruppen mit pädagogisch tätigem Personal und 44 Interviews mit Kindern erhoben.

Die meisten Kinder äußern sich positiv zu Bewegung, Spiel und Sport im Ganztag. Mit den Aktivitäten verbinden sie vor allem Spaß und Freude, aber auch sich sportlich zu verbessern oder allgemein etwas zu lernen, sind zentrale Motive. So sagt ein Kind: „Von Sport bekommen wir viel Kraft und Spaß. Und wenn wir Spaß und Kraft bekommen, dann haben wir keinen Ärger und von Sport können wir auch viel lernen“ [44, S. 166]. Daneben äußern sie aber auch Wünsche zur Verbesserung von Bewegungsmöglichkeiten in der Ganztagsgrundschule. Diese spiegeln sich in 4 Clustern wider: Material, Räume, Angebote und methodisch-didaktische Inszenierung. Die materiellen (z. B. Schaukel, Tunnelrutsche) und räumlichen Wünsche (z. B. Sportraum, Keller mit Rasen) zeigen sich in einer großen Vielfalt und Kreativität. Die Angebotswünsche reichen von spezifischen Sportarten über Bewegungsspiele bis hin zu informellen Bewegungs- und Spielmöglichkeiten und unterstreichen damit die große Bandbreite an kindlichen Interessen.

Im Bereich der methodisch-didaktischen Inszenierung stehen die Mitbestimmungsmöglichkeiten der Kinder im Fokus. Sie sehen diese oftmals auf die Auswahl von Spielen begrenzt. Einige Kinder wünschen sich, in die Gestaltung von Bewegungsangeboten im Ganztag eingebunden zu werden. Auf der anderen Seite werden auch Vorgaben durch die Erwachsenen eingefordert: „Also schon, dass wir ein bisschen bestimmen, aber auch nicht alles. Weil, das ist dann auch ein großes Chaos“ [44, S. 176].

Ebenso wie die meisten Kinder bewertet das pädagogische Personal Bewegung, Spiel und Sport im schulischen Ganztag positiv [45]. Dabei werden vielfältige Potenziale für das Aufwachsen von Kindern gesehen, vor allem für diejenigen, die außerhalb der Schule keinen Zugang zu Bewegungs- und Sportangeboten haben. Die positiven Assoziationen reichen von allgemeinen Zuschreibungen wie Spaß, Lernen, Ausgleich oder Bedürfnisbefriedigung bis hin zu konkreten entwicklungs- und gesundheitsfördernden Potenzialen. Diesbezüglich werden u. a. Persönlichkeitsentwicklung, Konzentrationsförderung, Sprachförderung oder die Stärkung sozialer Kompetenzen hervorgehoben.

Auf der anderen Seite sieht das Personal zahlreiche Herausforderungen in der Umsetzung von Bewegungs‑, Spiel- und Sportaktivitäten im Ganztag [45]. Neben mangelnden personellen, räumlichen und materiellen Ressourcen beklagen sie grundlegende Defizite in den Strukturen und Rahmenbedingungen sowie eine fehlende Wertschätzung für Bewegung, Spiel und Sport. Darüber hinaus geben die Befragten an, sich in bestimmten Bereichen mehr Unterstützung zu wünschen – etwa in der methodisch-didaktischen Gestaltung von Angeboten sowie bei den Themen Sicherheit, Heterogenität der Lerngruppen und individuelle Förderung. Auch Kooperations- und Vernetzungsmöglichkeiten im Sozialraum werden als relevante Themenfelder benannt [45].

Die aktuellen qualitativen Daten deuten auf zahlreiche Herausforderungen hin, die mit den Rahmenbedingungen für Bewegungsangebote in Verbindung stehen. Hier gilt es in weiteren Forschungsarbeiten anzuknüpfen, um differenzierte Erkenntnisse über die aktuelle Situation von Bewegung, Spiel und Sport im Ganztag zu generieren. Darauf aufbauend kann eine systematische Qualitätsentwicklung erfolgen, um eine individuelle, umfassende und hochwertige Bewegungsförderung im Ganztag zu sichern. Die Aufarbeitung der Forschungslücken erscheint besonders relevant, da Kinder ihre Freizeit zunehmend in der Schule verbringen und die Angebotsqualität als wesentlicher Faktor für die Entfaltung von Bildungs- und Entwicklungspotenzialen gilt [46].

## Qualitätsdimensionen für Bewegungs‑, Spiel- und Sportangebote im Ganztag

Als Grundlage für eine systematische Qualitätsentwicklung von Bewegungs‑, Spiel- und Sportangeboten im Ganztag sind zentrale Qualitätsindikatoren erforderlich. Dafür wurde in einem heuristischen Vorgehen ein Orientierungsrahmen entwickelt, der bislang nur teilweise empirisch abgesichert werden konnte [19]. Das Input-Prozess-Output-Modell zur Ganztagsschulqualität bildet mit den 3 Dimensionen Kontext/Input, Prozess und Wirkung die strukturgebende Grundlage [47]. Der Orientierungsrahmen umfasst 8 Ansatzpunkte:

1. *Konzept für den Ganztag*: In einem solchen Konzept sollten die Rahmenbedingungen für die Umsetzung von Bewegung, Spiel und Sport auf unterschiedlichen Ebenen festgelegt werden. Grundlegend ist dafür ein umfassendes Verständnis von Bewegungsförderung, das sowohl pädagogische als auch gesundheitsbezogene Perspektiven berücksichtigt.
2. *Kooperation und Vernetzung innerhalb des Sozialraums:* Ein gut funktionierendes Netzwerk mit den verschiedenen relevanten Partnern ist eine wesentliche Voraussetzung für die Gestaltung eines vielfältigen und an den Interessen der Kinder orientierten Angebots.
3. *Qualifikation des Personals*: Die Kompetenzen des Personals bilden für eine fachlich fundierte und pädagogisch-didaktisch gehaltvolle Umsetzung von Bewegung, Spiel und Sport im Ganztag das zentrale Fundament. Wie im Forschungsstand skizziert, bestehen umfassende Qualifizierungsbedarfe aufseiten des Personals [41, 45].
4. *Bewegungsräume und Materialien: *Sie besitzen einen hohen Stellenwert für eine umfassende und individuelle Bewegungsförderung. Als Lern- und Lebensort sollte der Ganztag über bewegungsfreundlich gestaltete Schulgebäude und -gelände sowie geeignete Materialien verfügen. Dadurch können insbesondere Bewegungsaktivitäten außerhalb von angeleiteten Angeboten maßgeblich unterstützt werden.
5. *Didaktische Leitidee zu den Angeboten:* Sie bildet eine zentrale Grundlage für die Umsetzung von Bewegung, Spiel und Sport und sollte daher auch in fachspezifischen Ganztagskonzepten an Grundschulen verankert sein. Der Doppelauftrag des Schulsports bildet den pädagogischen Rahmen für die Ausgestaltung, wobei die Interessen der Kinder im Mittelpunkt stehen sollten.
6. *Partizipation und Teilhabe der Kinder: *Eine Beteiligung der Kinder auf unterschiedlichen Ebenen ist für eine bedürfnisorientierte Ausrichtung des Bewegungs‑, Spiel- und Sportangebots von besonderer Bedeutung und trägt zur Qualitätsentwicklung im Ganztag bei.
7. *Individuelle Förderung:* Sie gilt in der Ganztagsschulentwicklung als zentrales Ziel und gewinnt angesichts der zunehmenden Teilnahme von Kindern am Ganztag an Bedeutung. Eine umfassende Bewegungsförderung, die sich an den Bedarfen, Voraussetzungen und Bedürfnissen der Kinder orientiert, reicht von Möglichkeiten zum freien Bewegungsspiel über kompensatorische Angebote und sportbezogene Arbeitsgemeinschaften bis hin zur Förderung von besonderen Begabungen.
8. *Evaluation:* Eine wissenschaftliche Begleitung ist ein wesentlicher Bestandteil der Qualitätssicherung von Bewegung, Spiel und Sport im Ganztag und für alle Fragen der Qualitätsentwicklung im Ganztag relevant. Dabei sind sowohl die Begleitung von Entwicklungsprozessen und die Erfassung der aktuellen Situation als auch die Untersuchung von Wirkungszusammenhängen von Bedeutung.

## Perspektiven für die Bewegungsförderung im Ganztag

Der Beitrag hat gezeigt, dass mit der Einführung der Ganztagsgrundschule neben dem verpflichtenden Sportunterricht in der Schule und dem freiwilligen Sport im Verein zusätzliche Bewegungsmöglichkeiten entstanden sind. Diese bieten insbesondere die Chance, auch Kinder aus sozial herausfordernden Lagen zu erreichen. Zugleich sind die Ganztagsangebote mit zahlreichen Herausforderungen verbunden, zum Beispiel fehlende finanzielle, räumliche und personelle Ressourcen, unzureichende Qualifikation des Personals sowie fehlende Qualitätsstandards. Dennoch werden Bewegungs‑, Spiel- und Sportaktivitäten im Ganztag sowohl von den beteiligten Kindern als auch vom pädagogischen Personal überwiegend positiv eingeschätzt. Aus gesundheitlicher Sicht bieten sie Gelegenheit für mehr Bewegungsaktivitäten über den Tag, aus pädagogischer Sicht bieten sie vielfältige Entwicklungschancen in individueller und sozialer Hinsicht. Um dieses Potenzial möglichst umfassend zu nutzen, sollten diese neuen Angebote der Bewegungsförderung mit den bestehenden Angeboten verknüpft werden.

Als konzeptionelle Basis für Bewegungsangebote im Ganztag bietet sich ein differenziertes Verständnis von Lernen an, das je nach Lernort, Lerninhalt und Inszenierung des Lernens unterschiedliche Arten des Lernens (Lernmodalitäten) unterscheidet [48]:

- Der Sportunterricht folgt überwiegend einem *formellen Lernbegriff*. Dieses Lernen findet im Rahmen zielgerichteter, strukturierter und verpflichtender Erziehungs- und Unterrichtsprozesse statt und wird in der Regel zertifiziert (z. B. durch die Sportnote).
- Bewegungs‑, Spiel- und Sportangebote im Ganztag sind überwiegend einem *nichtformellen Lernbegriff* zuzuordnen. Auch dieses Lernen ist zielgerichtet und geplant, die Teilnahme ist allerdings freiwillig und die Leistungen werden nicht zertifiziert.
- Nichtangeleitete Bewegungs‑, Spiel- und Sportaktivitäten können einem *informellen Lernbegriff *zugeordnet werden. Sie erfolgen weitgehend ungeplant, unorganisiert und freiwillig, beispielsweise auf dem Schulhof in den Pausen oder während der Betreuung.

Während der schulische Pflichtunterricht stärker auf die Entwicklung der Kinder ausgerichtet ist, können außerunterrichtliche Ganztagsangebote stärker an ihren Bedürfnissen ansetzen [49]. Das bedeutet nicht, dass der Sportunterricht die Bedürfnisse der Kinder unberücksichtigt lässt oder außerunterrichtliche Ganztagsangebote deren Entwicklungsförderung ausblenden; allerdings unterscheiden sich die Schwerpunktsetzungen [45].

Neben den angeleiteten Angeboten im unterrichtlichen und außerunterrichtlichen Bereich bieten informelle, nichtangeleitete Aktivitäten ein erhebliches Potenzial für die Alltagsbewegung von Kindern. Bewegungsfreundlich gestaltete Innen- und Außenräume ermöglichen es den Kindern, ihre freie Zeit im Ganztag selbstbestimmt für Bewegung zu nutzen. Gerade das freie Bewegungsspiel bietet über die motorische Aktivität hinaus vielfältige Lern- und Entwicklungsmöglichkeiten, die von Autonomie und selbstständigem Problemlösen bis hin zum Erwerb sozialer Kompetenzen reichen [18].

Die Vernetzung unterschiedlicher Aktivitäts- bzw. Angebotsformen kann damit nicht nur zu einem zeitgemäßen Bildungsangebot, sondern auch zu einer umfassenden Bewegungsförderung für alle Kinder beitragen. Ein Beispiel hierfür ist die Idee des „Bewegungsbands“, das verschiedene Bewegungsmöglichkeiten über den gesamten Schultag hinweg miteinander verbindet: vom bewegten Schulweg und einer bewegten Begrüßung am Morgen über Bewegungspausen im Unterricht, Bewegungsspiele in den Pausen und den Sportunterricht bis hin zu bewegungsorientierten Ganztagsangeboten am Nachmittag und selbstbestimmtem Bewegungsspiel in freien Ganztagszeiten [50].

## Fazit

Durch die Vernetzung der 3 Bewegungsfelder Sportunterricht, Ganztagssport sowie Vereinssport (Abb. 1) könnte sich die Ganztagsschule als Bildungslandschaft „im Kleinen“ etablieren. Voraussetzung ist allerdings, dass die Bedeutung von Bewegungs‑, Spiel- und Sportaktivitäten für die Entwicklung und Gesundheit junger Menschen von Bildungspolitik, Bildungsverwaltung und Bildungswissenschaft anerkannt und entsprechend gefördert wird. Auf diese Weise können Kinder für Bewegung, Spiel und Sport begeistert und langfristig an einen aktiven Lebensstil herangeführt werden.

## Data Availability

Für diesen Beitrag wurden keine Datensätze erstellt oder analysiert.
